# Critical Crossroads: The Vital Role of Timely Diagnosis in Severe Amyopathic Dermatomyositis

**DOI:** 10.7759/cureus.70875

**Published:** 2024-10-05

**Authors:** Ana Carolina Monteiro, Tomás Santana, Ana Rita Tomás, Catarina Negrao, Clara Matos

**Affiliations:** 1 Internal Medicine Department, Hospital Prof. Dr. Fernando Fonseca, Lisbon, PRT; 2 Radiology Department, Hospital CUF (Companhia União Fabril) Tejo, Lisbon, PRT

**Keywords:** amyopathic dermatomyositis, anti-mda5 antibodies, dermatomyositis, interstitial lung disease, pneumomediastinum

## Abstract

Clinically amyopathic dermatomyositis (CADM) is an uncommon subtype of dermatomyositis (DM) characterized by the typical cutaneous manifestations of DM but without clinical or enzymatic signs of muscle inflammation.

We report a case of a 61-year-old woman with a four-week history of dry cough, myalgias, chills, pleuritic chest pain, and worsening shortness of breath. She also had a five-year history of inflammatory polyarthralgia. Upon admission, she was hypoxemic and had subcutaneous emphysema, along with painful papules and erythematous lesions on her fingers. A thoracic computed tomography scan revealed pneumomediastinum and a chronic reticular interstitial pattern. Initially suspected of having COVID-19, laboratory results showed a negative COVID-19 test but positive anti-melanoma differentiation-associated gene 5 antibody (anti-MDA5), leading to a diagnosis of CADM. Treatment with prednisolone and mycophenolate mofetil was initiated, resulting in subsequent clinical improvement.

In conclusion, this case of anti-MDA5 positive CADM underscores the diverse range of clinical and radiological findings and the diagnostic challenges they pose. It highlights the importance of anti-MDA5 antibodies as a valuable diagnostic and prognostic tool, given their association with an elevated risk of developing interstitial lung disease (ILD), which may follow a rapidly progressive course and can be further complicated by pneumomediastinum.

## Introduction

Dermatomyositis (DM) is a rare inflammatory disorder characterized by distinctive skin lesions and bilateral symmetric proximal muscle weakness, often associated with increased serum skeletal muscle enzyme levels and myopathic alterations detectable through electromyography or muscle biopsy. Approximately 20% of patients with DM exhibit typical cutaneous manifestations but do not present with any clinical or enzymatic signs of muscle inflammation within six months of onset, leading to the diagnosis of clinically amyopathic dermatomyositis (CADM) [1].

Pulmonary manifestations are observed in 50% of patients diagnosed with CADM, with interstitial lung disease (ILD) being particularly prevalent. Patients with coexisting CADM and ILD commonly experience dyspnea or hypoxemia triggered by physical exertion, along with fever, cough, and reduced exercise tolerance [2].

CADM is an idiopathic autoimmune disorder associated with various autoantibodies, one of the most specific being anti-melanoma differentiation-associated gene 5 (anti-MDA5) antibodies. Interestingly, anti-MDA5 positive patients exhibit a significantly higher likelihood, approximately 20 times greater, of developing rapidly progressive interstitial lung disease (RP-ILD) compared to seronegative patients [2]. Thus, this diagnostic tool aids in identifying individuals with DM who are at risk of rapidly developing ILD, with both high sensitivity and specificity [2]. We report a case of anti-MDA5 positive CADM with complex pulmonary involvement.

## Case presentation

We report the case of a 61-year-old woman with no relevant medical history who presented with a four-week history of dry cough with bloody sputum, myalgias, chills, anorexia, diffuse pleuritic chest pain, and progressively worsening physical activity tolerance, culminating in severe dyspnea at rest. Additionally, she had a five-year history of additive polyarthralgia affecting the knees, ankles, elbows, wrists, and hands in an asymmetrical pattern. Her joint pain was most severe in the mornings and was alleviated by movement and chronic use of analgesics such as paracetamol and ibuprofen. It was accompanied by stiffness that generally persisted for approximately 45 minutes.

Upon admission, the patient was alert, with a tympanic temperature of 37.9 ºC. She was hemodynamically stable but had a respiratory rate of 25 breaths per minute and an oxygen saturation level of 85% while breathing room air. Subcutaneous emphysema was noted in the paratracheal and supraclavicular regions, and crackling rales were heard in both lower lung fields. The physical exam also revealed small, painful papules on the extensor surfaces of her fourth and fifth fingers, along with punctiform, non-confluent erythematous lesions on the palmar surfaces of her hands and fingers, which were slightly tender to the touch. Despite these findings, no muscle strength deficits were observed, and the Gowers sign was negative.

Initial laboratory tests revealed inflammatory anemia and elevated inflammatory biomarkers, while arterial blood gas analysis showed hypoxemia and hypocapnia (Table 1).

**Table 1:** Initial laboratory investigation

Laboratory tests	Patient values	Reference range
Hemoglobin	11.4 g/dL	12.1–15.1 g/dL
White blood cells	4.7 × 10^3^/µL	4.0–11.0 × 10^3^/µL
Platelets	170 × 10^3^/µL	150–450 × 10^3^/µL
Alanine transaminase	35 U/L	7–56 U/L
Aspartate transaminase	45 U/L	5–40 U/L
Alkaline phosphatase	107 U/L	44–147 U/L
γ-Glutamyl transpeptidase	36 U/L	<48 U/L
Total bilirubin	0.8 mg/dL	0.3–1.0 mg/dL
Lactate dehydrogenase	178 U/L	140–280 U/L
Serum creatinine	0.9 mg/dL	0.6–1.3 mg/dL
Blood urea nitrogen	11.8 mg/dL	7–20 mg/dL
Sodium	142 mmol/L	135–145 mmol/L
Potassium	4.2 mmol/L	3.5–5.0 mmol/L
Creatinine kinase	43 U/L	56–244 U/L
Aldolase	8.0 U/L	1.5–8.1U/L
C-reactive protein	5.3 mg/dL	<0.30 mg/dL
Erythrocyte sedimentation rate	54 mm/h	0-20 mm/h
Ferritin	>2000 ng/mL	24–307 ng/mL
Arterial blood gas analysis (room air):		
pH	7.48	7.35–7.45
pCO_2_	30	35–45 mmHg
pO_2_	55	>60–100 mmHg
HCO_3_	20	22–26 mEq/L
SatO_2_	88	>94%
Lactate	1.1	<2 mmol/L

**Figure 1:**
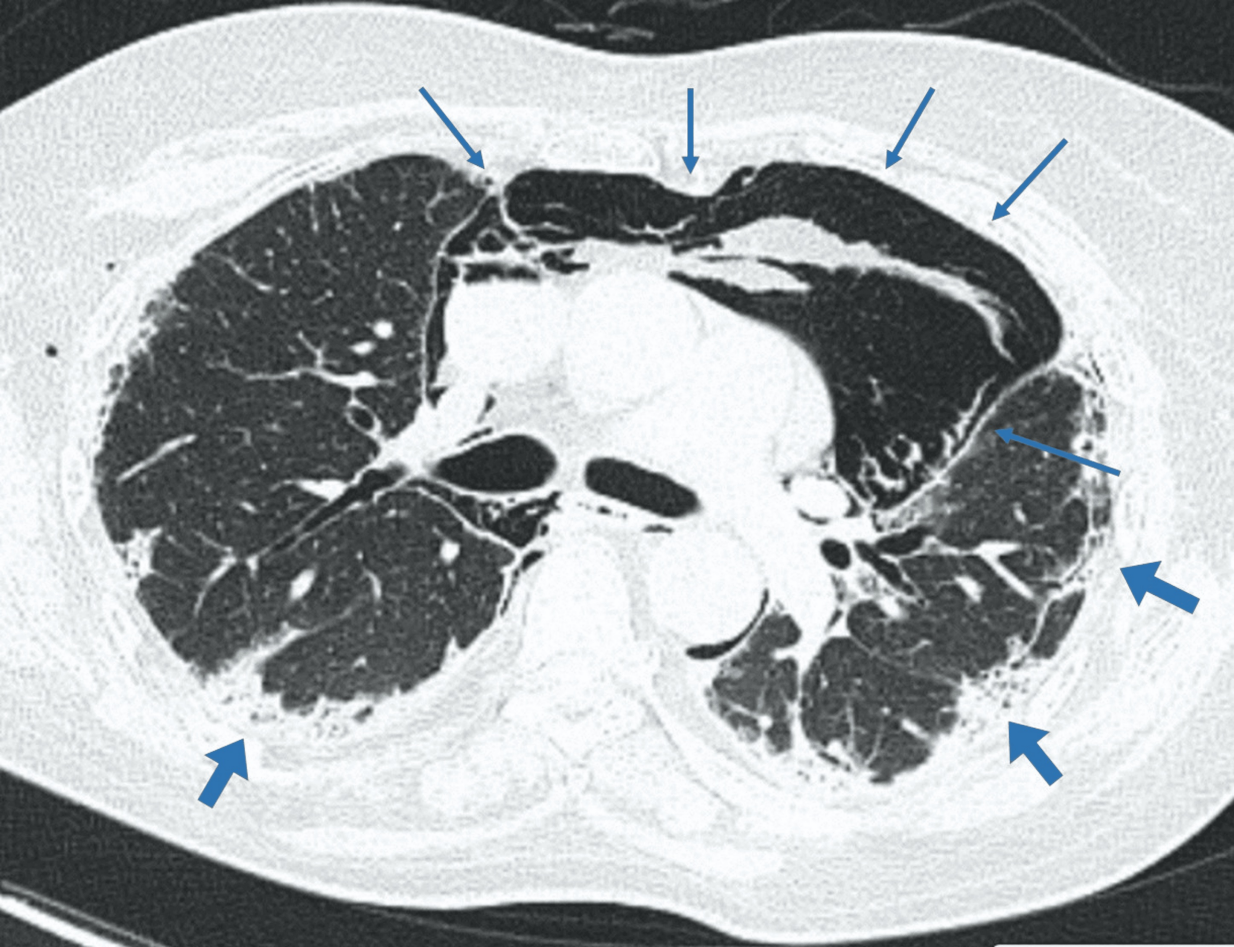
Initial chest CT scan Chest CT image showing an extensive pneumomediastinum (thin arrows) and some small areas of subcutaneous emphysema. The lung parenchyma presented thickening of the interlobular septa alongside bronchiectasis with a peripheral and basal predominance. Some of these areas were surrounded by ground-glass opacities and consolidations (thick arrows).

A chest computed tomography (CT) scan revealed extensive pneumomediastinum and areas of subcutaneous emphysema. The lung parenchyma showed thickening of the interlobular septa along with bronchiectasis, predominantly in peripheral and basal regions. Some of these areas were surrounded by consolidations and ground-glass opacities (Figure 1).

Given the global SARS-CoV-2 pandemic at that time, COVID-19 pneumonia complicated by spontaneous pneumomediastinum was initially considered the most likely diagnosis.

However, further diagnostic testing revealed negative results for all SARS-CoV-2 polymerase chain reaction tests. A comprehensive infectious and autoimmune workup returned negative results, except for the presence of antinuclear antibodies and a positive result for anti-melanoma differentiation-associated gene 5 antibody (anti-MDA5) (Table 2). The dermatological evaluation determined that the lesions on the fingers were characteristic of vasculitis or thrombosis.

**Table 2:** Subsequent investigation results

Laboratory investigation	Patient result
Blood culture	Negative
Sputum culture	Negative
SARS-COV-2 polymerase chain reaction tests	Negative
Serum protein electrophoresis	Polyclonal hypergammaglobulinemia
Human Immunodeficiency Virus antibodies	Non detectable
Interferon-gamma release assay test	Non detectable
Antinuclear antibody	1:320, mottled pattern
Anti-Ro antibody	Negative
Anti-La antibody	Negative
Anti-Sm antibody	Negative
Anti-Scl-70 antibody	Negative
Anti-RNP antibody	Negative
Anti Jo-1 antibody	Negative
Anti-Mi-2 antibody	Negative
Anti-Transcriptional intermediary factor (Anti-TIF) 1γ antibody	Negative
Anti-melanoma differentiation-associated gene 5 antibody (Anti-MDA5)	Positive

The final diagnosis of anti-MDA5 antibody-positive CADM) with cutaneous, articular, and pulmonary involvement was established.

A comprehensive screening, including an abdominopelvic CT scan, bone scintigraphy, upper endoscopy, colonoscopy, mammography, and ultrasounds of the breast, gynecological, and cervical regions, was conducted. No signs of cancer were detected. Pulmonary function tests revealed moderate restrictive ventilatory changes with reduced alveolar-capillary transfer capacity for carbon monoxide.

**Figure 2:**
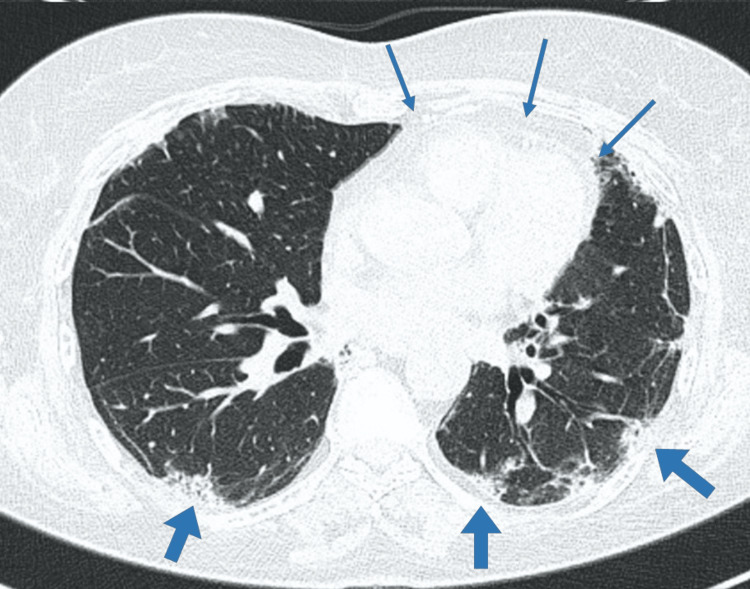
Re-evaluation chest CT scan No pneumomediastinum or subcutaneous emphysema were found (thin arrows). The previously described pulmonary chronic reticular interstitial pattern remained globally stable (thick arrows), now being interpreted as chronic fibrotic changes related to the underlying interstitial lung disease.

Treatment with prednisolone 1 mg/kg/day and mycophenolate mofetil 1 g/day was initiated. The patient showed noticeable clinical improvement, though moderate dyspnea persisted, and oxygen was still required upon exertion. A follow-up thoracic CT scan showed no pneumomediastinum or subcutaneous emphysema; the previously described chronic reticular interstitial pattern remained stable and was now interpreted as chronic fibrotic changes related to the underlying interstitial lung disease (Figure 2).

The patient was discharged from the hospital after a one-month stay, following favorable clinical and radiological outcomes. At the follow-up appointment, one month after discharge, the patient reported improvement in skin lesions, myalgias, fatigue, and cough. However, she continued to experience fatigue, particularly during moderate exertion, requiring a pause after walking approximately 200 meters to recuperate. During the physical examination, the patient was observed to be breathing comfortably at rest with a peripheral oxygen saturation of 98%. Pulmonary auscultation revealed scattered crackles, predominantly at the lung bases. She also had hypopigmented lesions on the upper limbs and hands, suggestive of previous scarring. There were no functional movement restrictions, and the Gowers sign was negative.

## Discussion

We report a case of anti-MDA5 positive CADM with pulmonary involvement. Our patient exhibited respiratory symptoms that could be attributed to coexisting CADM-associated ILD, and its rapid progression was consistent with anti-MDA5 positivity.

Spontaneous pneumomediastinum and subcutaneous emphysema are very rare complications of DM-associated lung disease [1]. More than half of pneumomediastinum cases in CADM patients are associated with heightened clinical aggressiveness [3]. It typically manifests within the first year following diagnosis and is rarely observed as an initial presentation [3].

Risk factors for developing pneumomediastinum include the presence of ILD, cutaneous vasculopathy, prior systemic corticosteroid use, young age, minimal elevation of creatine kinase, detectable anti-MDA5, and prior reductions in vital capacity and carbon monoxide diffusing capacity [3]. Our patient exhibited nearly all of these risk factors.

High-resolution chest CT and pulmonary function tests are crucial for diagnosing and assessing the severity of CADM-related ILD [4]. This case occurred during the COVID-19 pandemic, which complicated the differential diagnosis due to the similarities between the HRCT findings of CADM-associated rapidly progressive ILD and COVID-19 pneumonia, including ground-glass opacities, reticular patterns, and septal thickening [4]. Despite progressive respiratory symptoms and articular involvement that suggested an underlying multisystemic disorder, our patient was initially misdiagnosed with viral pneumonia.

Cancer screening should be performed at the time of diagnosis and regularly for the first three years, even if initial results are negative. CADM patients have a similar age-related malignancy risk as classic DM patients, with the greatest risk occurring within the first year after diagnosis [2].

Early and aggressive treatment with high doses of systemic corticosteroids combined with immunosuppressive agents is recommended for treating CADM-associated rapidly progressive ILD to reduce the risk of complications such as pneumomediastinum [5]. This approach, along with supportive measures (such as oxygen therapy, analgesic medication, and bed rest) and careful observation, is associated with a more favorable prognosis for CADM-related pneumomediastinum. Given the elevated surgical risks in patients with connective tissue disease-related ILD, conservative management is often preferred [5].

Pulmonary involvement is the leading cause of mortality in ADM, with severe ILD and pneumomediastinum being the most critical and potentially life-threatening complications. While pneumomediastinum can be fatal, the primary contributor to mortality is often the presence, rapid progression, and severity of ILD [4]. In fact, CADM patients with ILD experience earlier deterioration, with a six-month survival rate of 60%, compared to 80% for those without ILD [1].

Learning points

CADM is an uncommon variant of DM that presents a substantial diagnostic challenge due to its low prevalence and its diverse, non-specific, and often misleading clinical manifestations, such as the absence of muscle involvement that is characteristic of classic DM.

Pulmonary involvement, often associated with the presence of detectable anti-MDA5 antibodies, significantly impacts the overall prognosis as it can lead to potentially life-threatening complications, such as rapidly progressive diffuse ILD and pneumomediastinum.

Prompt recognition of this condition is crucial for the timely initiation of aggressive immunosuppressive treatment, as well as for appropriate malignancy and ILD surveillance.

## Conclusions

In conclusion, a comprehensive medical history and meticulous physical examination are essential for promptly considering the diagnosis of CADM, particularly when respiratory, cutaneous, and articular manifestations are present. This approach is vital not only for the timely initiation of aggressive treatment but also for the implementation of appropriate ILD and malignancy surveillance, helping to prevent further morbidity and mortality in affected patients.
